# Immortalized Human Schwann Cell Lines Derived From Tumors of Schwannomatosis Patients

**DOI:** 10.1371/journal.pone.0144620

**Published:** 2015-12-14

**Authors:** Kimberly Laskie Ostrow, Katelyn Donaldson, Jaishri Blakeley, Allan Belzberg, Ahmet Hoke

**Affiliations:** 1 Department of Neurology, The Johns Hopkins School of Medicine, Baltimore, MD, 21205, United States of America; 2 Deparment of Oncology, The Johns Hopkins School of Medicine, Baltimore, MD, 21205, United States of America; 3 Department of Neurosurgery, The Johns Hopkins School of Medicine, Baltimore, MD, 21205, United States of America; 4 Department of Neuroscience, The Johns Hopkins School of Medicine, Baltimore, MD, 21205, United States of America; University of North Carolina School of Medicine, UNITED STATES

## Abstract

Schwannomatosis, a rare form of neurofibromatosis, is characterized predominantly by multiple, often painful, schwannomas throughout the peripheral nervous system. The current standard of care for schwannomatosis is surgical resection. A major obstacle to schwannomatosis research is the lack of robust tumor cell lines. There is a great need for mechanistic and drug discovery studies of schwannomatosis, yet appropriate tools are not currently available. Schwannomatosis tumors are difficult to grow in culture as they survive only a few passages before senescence. Our lab has extensive experience in establishing primary and immortalized human Schwann cell cultures from normal tissue that retain their phenotypes after immortalization. Therefore we took on the challenge of creating immortalized human Schwann cell lines derived from tumors from schwannomatosis patients. We have established and fully characterized 2 schwannomatosis cell lines from 2 separate patients using SV40 virus large T antigen. One patient reported pain and the other did not. The schwannomatosis cell lines were stained with S100B antibodies to confirm Schwann cell identity. The schwannomatosis cells also expressed the Schwann cell markers, p75NTR, S100B, and NGF after multiple passages. Cell morphology was retained following multiple passaging and freeze/ thaw cycles. Gene expression microarray analysis was used to compare the cell lines with their respective parent tumors. No differences in key genes were detected, with the exception that several cell cycle regulators were upregulated in the schwannomatosis cell lines when compared to their parent tumors. This upregulation was apparently a product of cell culturing, as the schwannomatosis cells exhibited the same expression pattern of cell cycle regulatory genes as normal primary human Schwann cells. Cell growth was also similar between normal primary and immortalized tumor cells in culture. Accurate cell lines derived directly from human tumors will serve as invaluable tools for advancing schwannomatosis research, including drug screening.

## Introduction

Schwannomatosis (SWN), a rare form of neurofibromatosis characterized by the development of multiple benign schwannomas. Schwannomatosis is estimated to affect 1 in 40,000 people. However, given the increasing understanding of the phenotypic heterogeneity of this disorder, its true incidence is unknown. SWN differs from Neurofibromatosis Type 2 in that patients do not develop vestibular tumors. Schwannomatosis patients also do not harbor germline mutations in the merlin gene, NF2. [1–3], although their individual tumors are bi-allelically inactivated at NF2. Interestingly each tumor from a single schwannomatosis patient typically carries an unrelated mutation [4]. Germline alterations in the SWI/SNF-Related Matrix-Associated Protein (SMARCB1/INI1) gene [5, 6] and more recently in the Leucine-Zipper-Like Transcription Regulator 1(LTZR1) have been implicated in familial schwannomatosis cases [7]. Yet, two-thirds of schwannomatosis patients have no family history of disease [2]. Therefore, the development of multiple sporadic schwannomas cannot be completely explained by these known tumor suppressor genes. Additional research is required to decipher the cause of these tumors.

Thus far there has been very limited research focused on schwannomatosis, in part because it is considered a rare disease, in part because there have been limited resources dedicated to the syndrome and most importantly because a relevant cell line model of SWN has been lacking. No phenotypically & physiologically relevant screening systems for drug discovery or drug re-purposing are currently available for the schwannomatosis research community. Currently, surgical resection persists as the standard of care for schwannomatosis. It is therefore critical to develop research tools to elucidate the genetic basis of schwannoma tumorigenesis and to identify novel therapeutic agents. With no commercially available schwannomatosis cell lines, the need has arisen to generate a method to dissociate and produce high-purity Schwann cell cultures from patient tumor specimens in order to advance peripheral nerve sheath tumor treatment options.

Schwann cells dissociated from the sciatic nerve of SMARCB1/INI1 knockout mice have been used as an in vitro model of schwannomatosis. Given the complex genetics and supporting cell types that make up a schwannoma, however, this may not be an accurate disease model. Schwannomatosis tumors have been difficult to grow in culture as they are benign cells that do not proliferate rapidly and survive only a few passages before senescence. Our lab has extensive experience in establishing primary Schwann cell cultures, from rat, mouse and human. We have established immortalized human Schwann cell lines using hTERT and SV40 large T antigen, which retained phenotype after immortalization [8]. Here we describe the establishment of cell lines from human schwannoma tumors surgically removed from schwannomatosis patients with sporadic schwannomatosis. The cell lines retain essential genotype and phenotype characteristics after passaging and immortalization.

## Results

**Fig 1:**
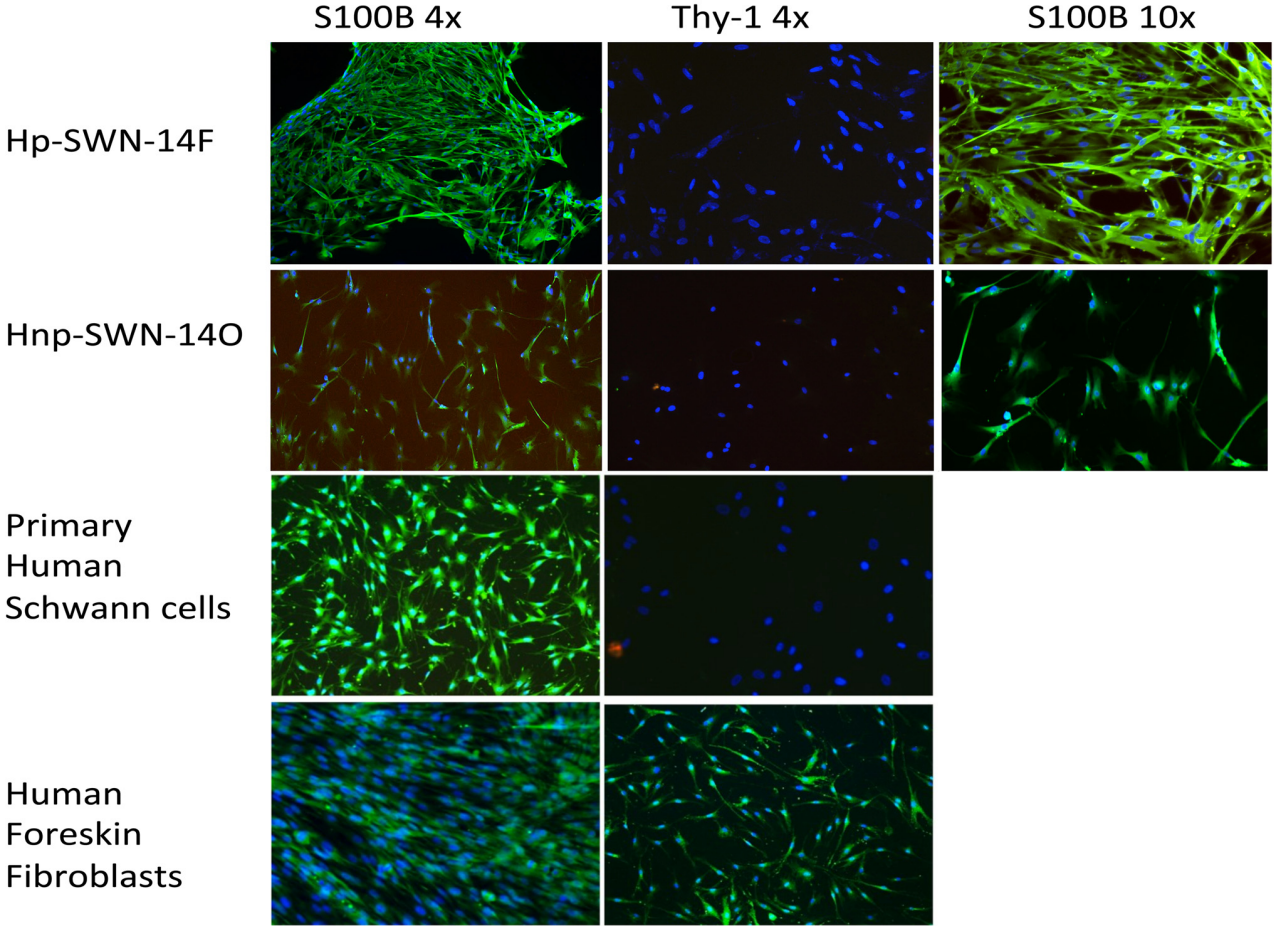
Immunocytochemistry of cell lines Schwannomatosis cell lines express the Schwann cell marker S100B and do not express the fibroblast marker Thy-1. Fibroblast cells stain positive for Thy-1. Green is a positive antibody test; blue is a DAPI nuclear stain.

**Fig 2:**
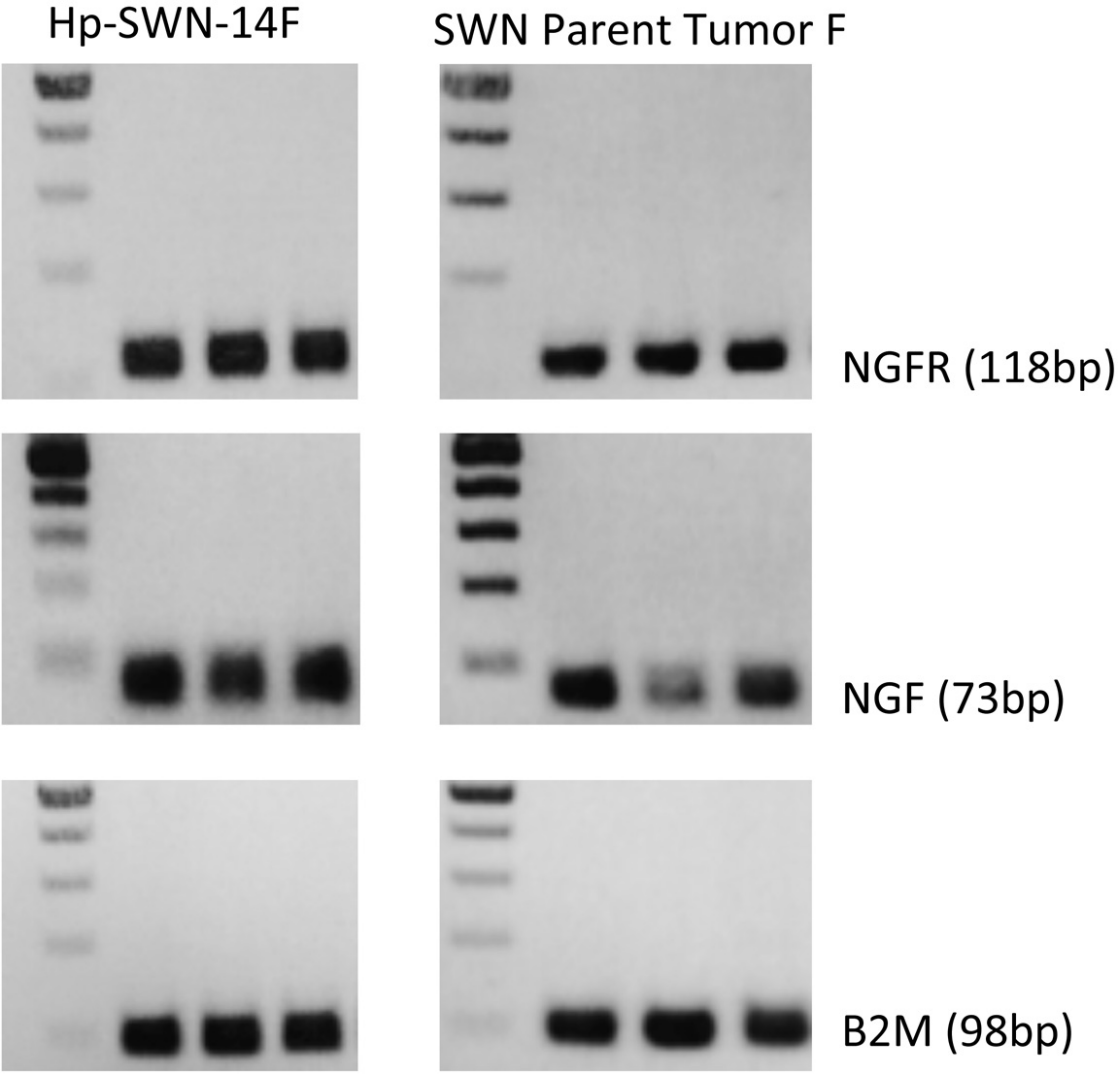
Schwannomatosis Cell line and Parent Tumor characteristics. Schwannomatosis cell line Hp-SWN-14F and its parent tumor s express Schwann cell markers, p75/NGFR, S100B, and NGF as determined by RT-PCR.

**Fig 3:**
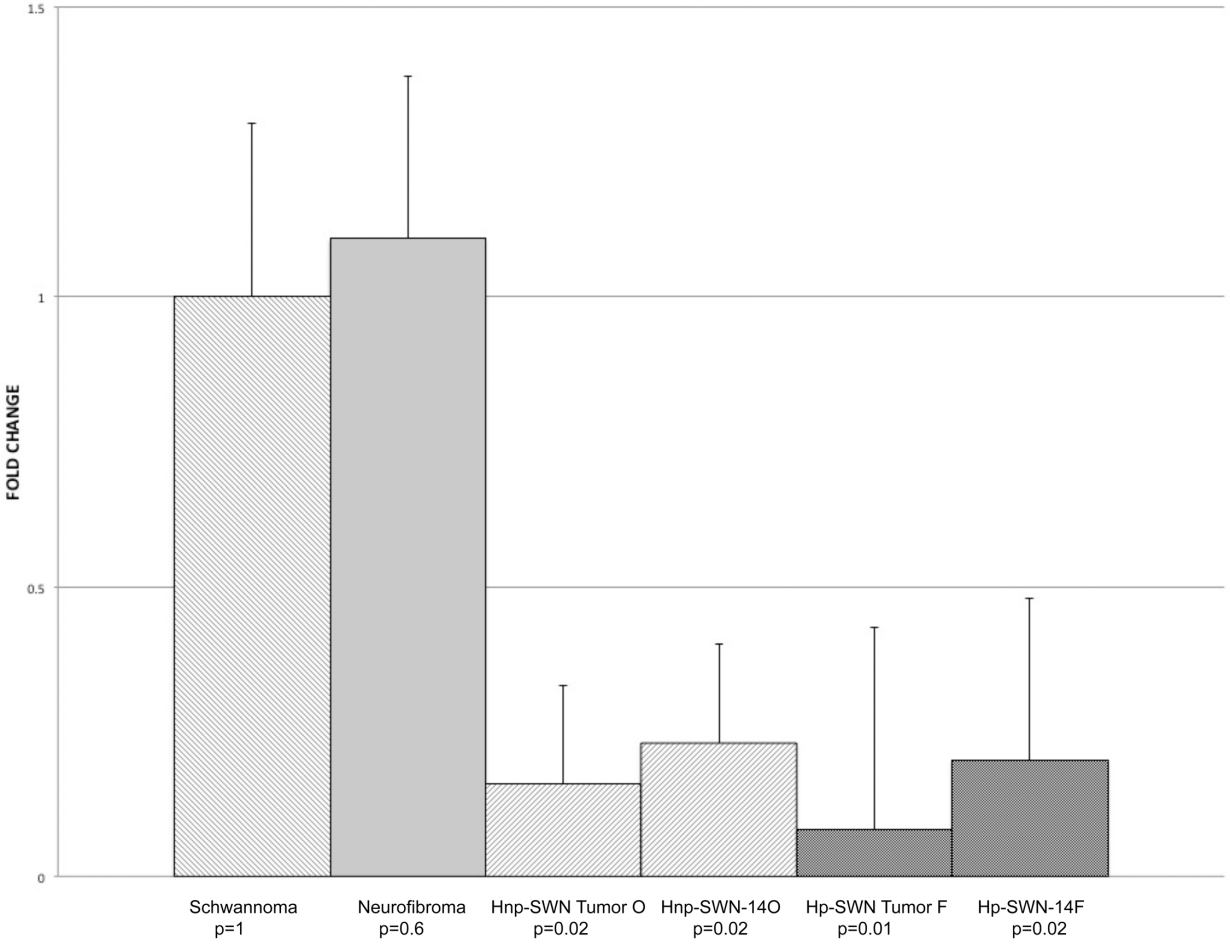
SMARCB1 expression in Parental Schwannomatosis Tumors. qRT-PCR was used to examine expression of SMARCB1 in cell lines and tumors. SMARCB1 expression was downregulated 8 fold in painful schwannomatosis tumor and 5 fold in the non-painful schwannomatosis tumor compared to a non-schwannomatosis schwannomas and a neurofibroma Hnp-SWN-14O and Hp-SWN-14F showed similar expression of SMARCB1 to the parent tumors from which they were derived.

We obtained schwannoma tissue specimens from patients with well-characterized clinical cases of schwannomatosis from surgical resection performed in the Comprehensive Neurofibromatosis (NF) Center at Johns Hopkins Hospital. We established 2 cells lines from separate schwannomatosis (SWN) patients. One tumor caused the patient great pain (Hp-SWN-14F) and the other was removed due to neurological deficit but was not associated with pain (Hnp-SWN-14O). The cell lines were established in our laboratory using standard cell culturing techniques, with minor modifications. In brief, tumor specimens were collected directly in the operating room in a vial of ice cold saline. The tissue was immediately processed by manual dissociation by mincing with a scalpel. The tissue was kept in cold saline while processing. The tissue was then incubated in L-15 media with collagenase and dispase for 3 hours to dissociate the cells. The cells were filtered through a 70um filter and seeded on plates coated with laminin. The cells were grown to confluency in T-25 flasks. After passaging the cells were examined by immunocytochemistry for the Schwann cell marker S100B, and the fibroblast marker Thy-1. The SWN cells were S100B positive. Fibroblast contamination was ruled out by a lack of Thy-1 immunostaining (Fig 1). At passage 3 the SWN cells were transfected with a lentivirus encoding SV40 large T antigen. Authentication of the cell line using Short Tandem Repeat (STR) profiling was employed to ensure the proper identity of the subsequent passages (S1 Fig). Schwann cell identity was also confirmed by qRT-PCR for Schwann cell markers (Fig 2). Both the parental tumors and the cell lines expressed transcripts for S100B, p75/NGFR, and NGF. We also compared SMARCB1 levels in the parental schwannomatosis tumors with those in tumors isolated from patients with other types of nerve sheath tumors. Both schwannomatosis–related tumors exhibited a significantly lower level of SMARCB1 expression, as compared to tumors isolated from patients with either single schwannomas or neurofibromas (Fig 3). Still, SMARCB1 levels were similar between the parent tumors and the corresponding schwannomatosis cell lines, further supporting retention of phenotype.

**Fig 4:**
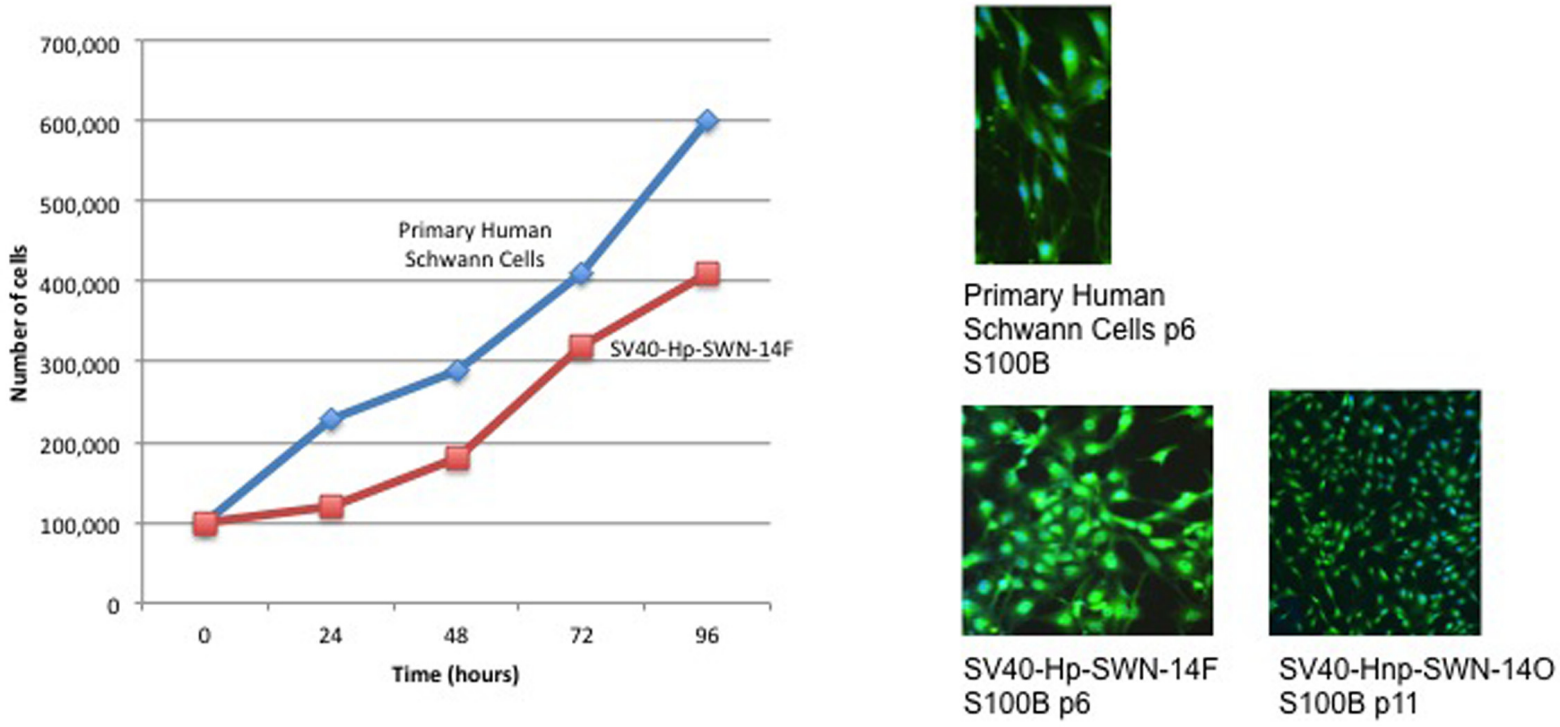
Schwannomatosis cell lines maintain Schwann cell characteristics after passaging and freezing. Primary Human Schwann cells and immortalized Hp-SWN-14F (at passage 6 exhibit similar growth rates and expression of S100B. Hnp-SWN-14O retains S100B staining and Schwann cell properties at passage 11.

As of the submission of this manuscript, the SWN cells have been passaged 11 times since immortalization with SV40. Total passaging since initial dissociation is 14. Primary Human Schwann cells, and schwannomatosis cell lines (Hp-SWN-14F and Hnp-SWN-14O) all positively stained for S100B after multiple passages and freeze-thaw cycles. (Fig 4). A potential concern with using immortalization by SV40 is the potential for increased growth rate and genomic changes, which alters the phenotype and therefore the value of the cell line as a research tool. To ensure the utility of these cells for examining growth inhibitors for drug testing, we performed a growth curve for each cell line. At passage 6 we performed a growth curve for Hp-SWN-14F schwannomatosis cell and non-immortalized human Schwann cells in culture. Population doubling time was similar in all cells (Fig 4).

**Fig 5:**
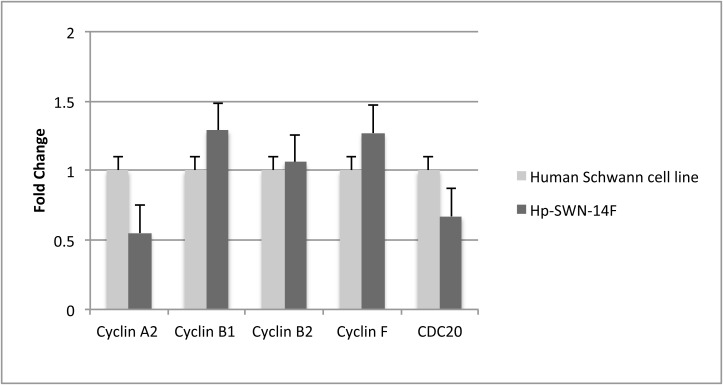
Expression of cell cycle regulators in SWN cell lines and primary human Schwann cells in culture. Several cell cycle related genes were differentially expressed in a SWN cell line compared with parent tumor by microarray. qPCR comparison of Primary Human Schwann Cells and Immortalized Hp-SWN-14F cell line suggest that the alterations in expression are due to active growth during cell culturing.

We also compared gene expression in the immortalized SWN cell lines (passage 6) and their parent tumors. The Illumina HT-12 microarray was used for gene expression analysis. This microarray platform contains probes for 47,000 transcripts and splice variants, with 31,000 annotated genes represented. This assay revealed some differences in gene expression between parent tumor cells and their respective immortalized cell lines. 509/31,000 (1.6%) of genes showed a 2 fold difference in the immortalized cell line when compared to the parent tissue. (S1 Table). Using DAVID we performed gene ontology clustering on the differentially expressed genes. The upregulated genes clustered into 12 groups. The cluster with greatest enrichment score contained mitosis related genes (Table 1). The downregulated genes clustered into 25 groups. The cluster with the greatest enrichment score contained genes relating to immune response and myelination (Table 2). We then compared gene expression of our immortalized SWN cell lines to that of normal primary human Schwann cells in culture. We focused on the 510 genes that were differentially expressed in SWN cells versus their parent tumor tissue. Of the 510 genes, 471 (92%) were expressed at a similar level in immortalized SWN cell lines and normal primary Human Schwann cell cultures (S1 Table). Several cell cycle related genes were differentially expressed in the schwannomatosis cell lines versus parent tumors by microarray. We examined these genes by qPCR of normal primary Human Schwann Cells versus immortalized SWN cell lines and detected no differences between the cells in culture, thus demonstrating that the alterations in expression are due to active growth during cell culturing (Fig 5).

**Table 1:** Gene Ontology of genes upregulated in cell lines compared to parent tumor tissue.

**Annotation Cluster 1: Enrichment Score: 30.374**	
**Term**	**Genes (35)**
GO:0000280~nuclear division	KIF23, CCDC99, NEK2, ANLN, AURKA,
GO:0007067~mitosis	KIF2C, CDCA8, TUBB, NCAPG, OIP5,
GO:0000087~M phase of mitotic cell cycle	NUSAP1, CDC20, BIRC5, CENPE, PBK,
GO:0048285~organelle fission	CDCA3, CCNF, TPX2, CENPF, CCNB1,
	CEP55, AURKB, PTTG1, FAM83D,
	ZWILCH, CCNA2, ASPM, CCNB2
	CDK2, SMC4, NCAPD2, MAD2L1
	FBXO5,UBE2C, ZWINT
**Annotation Cluster 2: Enrichment Score: 4.741**	
**Term**	**Genes (5)**
GO:0006268~DNA unwinding during replication	MCM7, MCM2, MCM4, HMGA1, MCM6
GO:0032392~DNA geometric change	
GO:0032508~DNA duplex unwinding	
**Annotation Cluster 3: Enrichment Score: 2.441**	
**Term**	**Genes (3)**
GO:0040001~establishment of mitotic spindle localization	CCDC99, CENPA, NUSAP1
GO:0051653~spindle localization	
GO:0051293~establishment of spindle localization	
**Annotation Cluster 4: Enrichment Score: 2.049**	
**Term**	**Genes (3)**
GO:0045841~negative regulation of mitotic meta/anaphase	MAD2L1, CENPF, CENPE
GO:0007094~mitotic cell cycle spindle assembly	
GO:0045839~negative regulation of mitosis	
GO:0051784~negative regulation of nuclear division	
GO:0030071~regulation of mitotic meta/anaphase transition	
GO:0010948~negative regulation of cell cycle process	
**Annotation Cluster 5: Enrichment Score: 2.046**	
**Term**	**Genes (5)**
GO:0051438~regulation of ubiquitin-protein ligase activity	CCNB1, MAD2L1, FBXO5, CDC20,
GO:0051340~regulation of ligase activity	UBE2C
GO:0031396~regulation of protein ubiquitination	
**Annotation Cluster 6: Enrichment Score: 1.514**	
**Term**	**Genes (4)**
GO:0051352~negative regulation of ligase activity	MAD2L1, FBXO5, CDC20, UBE2C
GO:0051444~negative regulation of ubiquitin-protein ligase activity	
GO:0031397~negative regulation of protein ubiquitination	
**Annotation Cluster 7: Enrichment Score: 1.431**	
**Term**	**Genes (4)**
GO:0051443~positive regulation of ubiquitin-protein ligase	CCNB1, FBXO5, CDC20, UBE2C
GO:0051351~positive regulation of ligase activity	
GO:0031398~positive regulation of protein ubiquitination	
**Annotation Cluster 8: Enrichment Score: 1.272**	
**Term**	
GO:0007044~cell-substrate junction assembly	**Genes (3)**
GO:0034329~cell junction assembly	DLC1, ITGA5, ACTN1
GO:0034330~cell junction organization	
**Annotation Cluster 9: Enrichment Score: 1.201**	
**Term**	
GO:0001556~oocyte maturation	**Genes (3)**
GO:0048599~oocyte development	CCNB1, FBXO5, TRIP13
GO:0009994~oocyte differentiation	
GO:0048477~oogenesis	
**Annotation Cluster 10: Enrichment Score: 1.165**	
**Term**	
GO:0031401~positive regulation of protein modification	**Genes (6)**
GO:0032270~ regulation of cellular protein metabolics	CCNB1, DLC1, CAV1, FBXO5, CDC20
GO:0051247~positive regulation of protein metabolics	UBE2C
**Annotation Cluster 11: Enrichment Score: 1.137**	
**Term**	
GO:0031400~negative regulation of protein modification	**Genes (5)**
GO:0032269~ regulation of cellular protein metabolic	CAV1, MAD2L1, FBXO5, CDC20
GO:0051248~negative regulation of protein metabolic	UBE2C
**Annotation Cluster 12: Enrichment Score: 1.025**	
**Term**	
GO:0006511~ubiquitin-dependent protein catabolic process	**Genes (4)**
GO:0010498~proteasomal protein catabolic process	CCNB1, MAD2L1, CDC20, UBE2C

**Table 2:** Gene Ontology of genes downregulated in cell lines compared to parent tumor tissue.

**Annotation Cluster 1: Enrichment Score: 6.778**	
**Term**	**Genes (12)**
GO:0002449~lymphocyte mediated immunity	C1QA, C1QB, SLC11A1, C3, FCER1G, C1R,
GO:0002250~adaptive immune response	SERPING1, C1S, C1QC, HLA-DMA, CD74, HLA-DRA
GO:0002460~adaptive immune response based on somatic recombination	
of immune receptors built from immunoglobulin superfamily domains	
**Annotation Cluster 2: Enrichment Score: 4.178**	
**Term**	**Genes (9)**
GO:0006956~complement activation	C1QA, C1QB, C3, C1R, SERPING1, C1S, VSIG4, CFD, C1QC
GO:0002541~activation of plasma proteins involved in acute inflammatory response	
GO:0051605~protein maturation by peptide bond cleavage	
GO:0016485~protein processing	
GO:0051604~protein maturation	
**Annotation Cluster 3: Enrichment Score: 3.938**	
**Term**	**Genes (7)**
GO:0042552~myelination	CD9, PLP1, ERBB2, LGI4, MAL, PMP22, MBP
GO:0007272~ensheathment of neurons	
GO:0008366~axon ensheathment	
**Annontation Cluster 4: Enrichment Score: 3.168**	
**Term**	**Genes (14)**
GO:0048812~neuron projection morphogenesis	NDN, ERBB3, PTPRZ1, ERBB2, CHST3, L1CAM,
GO:0048858~cell projection morphogenesis	GAS7, SLIT2, NRCAM, NCAM2, SEMA3B, RELN, FEZ1, GFRA3
**Annotation Cluster 5: Enrichment Score 3.130**	
**Term**	**Genes (4)**
GO:0002495~antigen processing and presentation of peptide antigen via MHC class II	FCER1G, HLA-DMA, CD74, HLA-DRA
GO:0002478~antigen processing and presentation of exogenous peptide antigen	
**Annotation Cluster 6: Enrichment Score: 3.050**	
**Term**	**Genes (13)**
GO:0007409~axonogenesis	NRCAM, NCAM2, NDN, PTPRZ1, ERBB3, ERBB2, CHST3
GO:0048667~cell morphogenesis involved in neuron differentiation	SEMA3B, L1CAM, RELN, SLIT2, GFRA3, FEZ1
GO:0000904~cell morphogenesis involved in differentiation	
**Annotation Cluster 7: Enrichment Score: 2.869**	**Genes (18)**
**Term**	PMP22, ATP1A2, LPAR1, SLC40A1, IFI6,
GO:0006873~cellular ion homeostasis	FXYD1, KCNMB4, PLP1, ERBB2, SNCA, MAL,MT3 CD9, SLC11A1, HAMP, APOE, LGI4, MBP,
GO:0055082~cellular chemical homeostasis	
GO:0050801~ion homeostasis	
	**Genes (6)**
**Annotation Cluster 8: Enrichment Score: 2.858**	CORO1A, PDGFB, CXCL16, S100A9
**Term**	ITGB2, SYK
GO:0030595~leukocyte chemotaxis	
GO:0060326~cell chemotaxis	
GO:0050900~leukocyte migration	**Genes (14)**
	NRCAM, TNS3, CORO1A, NDN, PDGFB, CXCL16, S100A9, ITGB2, RELN, ZEB2, TNFSF12, SLIT2, GFRA3, SYK
**Annotation Cluster 9: Enrichment Score: 2.312**	
**Term**	
GO:0016477~cell migration	**Genes (4)**
GO:0048870~cell motility	ALOX5AP, LTC4S, ALOX5, SYK
GO:0051674~localization of cell	
**Annotation Cluster 10: Enrichment Score: 2.161**	
**Term**	
GO:0019370~leukotriene biosynthetic process	
GO:0043450~alkene biosynthetic process	
GO:0006691~leukotriene metabolic process	**Genes (9)**
GO:0043449~cellular alkene metabolic process	LPL, PLP1, TBXAS1, PTGDS, ALOX5AP, LTC4S, ALOX5, CD74, SYK
**Annotation Cluster 11: Enrichment Score: 2.101**	
**Term**	
GO:0006633~fatty acid biosynthetic process	
GO:0046394~carboxylic acid biosynthetic process	**Genes (4)**
GO:0016053~organic acid biosynthetic process	PLEK, PDGFB, PDPN, APOE
GO:0008610~lipid biosynthetic process	
**Annotation Cluster 12: Enrichment Score: 2.099**	
**Term**	
GO:0010543~regulation of platelet activation	**Genes (10)**
GO:0030193~regulation of blood coagulation	NRCAM, METRN, LYN, CCND2, APOE, NTRK2, SLIT2, MBP, SPP1, MT3
GO:0050818~regulation of coagulation	
**Annotation Cluster 13: Enrichment Score: 1.996**	
**Term**	**Genes (11)**
GO:0050767~regulation of neurogenesis	SLC11A1, PDGFB, CCND2, ERBB2, TGFBR2, FPR1, ZEB2, RELN, LPAR1
GO:0051960~regulation of nervous system development	CD74, SYK
GO:0060284~regulation of cell development	
**Annotation Cluster 14: Enrichment Score: 1.851**	
**Term**	**Genes (7)**
GO:0045860~positive regulation of protein kinase activity	LYN, CCND2, HCLS1, TNFRSF14, IL34, FAM129A, SYK
GO:0033674~positive regulation of kinase activity	
GO:0051347~positive regulation of transferase activity	
**Annotation Cluster 15: Enrichment Score: 1.742**	
**Term**	
GO:0001934~positive regulation of protein amino acid phosphorylation	
GO:0042327~positive regulation of phosphorylation	
GO:0010562~positive regulation of phosphorus metabolic process	
GO:0045937~positive regulation of phosphate metabolic process	**Genes (7)**
GO:0031401~positive regulation of protein modification process	CD9, VWF, PLEK, PLSCR4, SERPING1, WAS, PROS1
**Annotation Cluster 16: Enrichment Score: 1.682**	
**Term**	
GO:0007596~blood coagulation	
GO:0050817~coagulation	**Genes (14)**
GO:0007599~hemostasis	PDGFB, C13ORF15, ERBB2, TGFBR2, FPR1, ZEB2,
GO:0050878~regulation of body fluid levels	LPAR1, CD74, SLC11A1, CCND2, APOE, RELN, SYK, DUSP6
**Annotation Cluster 17: Enrichment Score: 1.674**	
**Term**	
GO:0045859~regulation of protein kinase activity	**Genes (6)**
GO:0043549~regulation of kinase activity	CORO1A, LST1, ERBB2, TNFRSF14, VSIG4, SYK
GO:0051338~regulation of transferase activity	
**Annotation Cluster 18: Enrichment Score: 1.648**	**Genes (4)**
**Term**	LST1, ERBB2, TNFRSF14, VSIG4
GO:0050670~regulation of lymphocyte proliferation	
GO:0032944~regulation of mononuclear cell proliferation	
GO:0070663~regulation of leukocyte proliferation	
**Annotation Cluster 19: Enrichment Score: 1.636**	
**Term**	
GO:0032945~negative regulation of mononuclear cell proliferation	**Genes (11)**
GO:0050672~negative regulation of lymphocyte proliferation	EGR1, CEBPA, PLEK, LYN, HCLS1, IRF8, TGFBR2, HLA-DMA
GO:0070664~negative regulation of leukocyte proliferation	CD74, PIK3R1, SYK
**Annotation Cluster 20: Enrichment Score: 1.573**	
**Term**	**Genes (3)**
GO:0030097~hemopoiesis	APOE, APOC1, ABCA1
GO:0048534~hemopoietic or lymphoid organ development	
GO:0002520~immune system development	
**Annotation Cluster 21: Enrichment Score: 1.159**	
**Term**	
GO:0015914~phospholipid transport	**Genes (3)**
GO:0030301~cholesterol transport	APOE, APOC1, NFKBIA
GO:0015918~sterol transport	
GO:0042157~lipoprotein metabolic process	
**Annotation Cluster 22: Enrichment Score: 1.152**	
**Term**	**Genes (3)**
GO:0032374~regulation of cholesterol transport	CD86, TGFBR2, SYK
GO:0032371~regulation of sterol transport	
GO:0032368~regulation of lipid transport	
**Annotation Cluster 23: Enrichment Score: 1.129**	
**Term**	
GO:0046638~positive regulation of alpha-beta T cell differentiation	
GO:0046637~regulation of alpha-beta T cell differentiation	
GO:0046635~positive regulation of alpha-beta T cell activation	**Genes (5)**
	CORO1A, PLEK, GSN, SCIN, SPTBN1
**Annotation Cluster 24: Enrichment Score: 1.025**	
**Term**	
GO:0008064~regulation of actin polymerization or depolymerization	
GO:0030832~regulation of actin filament length	
GO:0032956~regulation of actin cytoskeleton organization	
	**Genes (3)**
**Annotation Cluster 25: Enrichment Score: 1.024**	SORBS1, ERBB3, PIK3R1
**Term**	
GO:0046326~positive regulation of glucose import	
GO:0010828~positive regulation of glucose transport	
GO:0046324~regulation of glucose import	
GO:0010827~regulation of glucose transport	

## Discussion

We have created human immortalized schwannomatosis cell lines derived from surgical resections of tumors from schwannomatosis patients. Previously reported cell culturing methods rely upon relatively harsh conditions to dissociate Schwann cells from the schwannoma tumor specimens. Frequently, trypsin is utilized as the predominant enzyme to break apart the tumor sample into its cellular components [9]. Some older protocols also include lengthy protease incubations that may extend overnight [10]. By comparison, the protocol used in our study involves gentle dissociation with dispase and collagenase over a shorter time period to increase cell survival rates. This method of dissociation generates high yield schwannoma cultures of a high purity. The cell lines are made up entirely of Schwann cells as demonstrated by S100B staining, gene expression analysis of the Schwann cell markers, NGF, NGFR, and S100B, and lack of anti-Thy-1 immunostaining.

It has been of utmost importance that our immortalized SWN cell lines retain phenotypic and genotypic characteristics of the tumor tissues from which they were derived. To this end we have fully characterized gene expression of both cell lines and their parent tumor. The non-painful SWN cell line (Hnp-SWN-14O) was derived from a patient harboring a mutation in SMARCB1. The expression level of SMARCB1 in both the tumor tissue and cell line was significantly downregulated when compared to non-SWN related schwannomas. The SWN tumor tissue and corresponding cell line exhibit similar SMARCB1 expression levels. Therefore the SWN cell line reflects the parental tumor regarding the expression of the key gene linked to schwannomatosis. Upon examination of gene expression in cell lines and their parent tumor by microarray analysis, we discovered some differences (1.6% difference between cell line and parental tissue). The genes downregulated in the SWN cell lines were related to immune response, and myelination. This was not surprising as the tumor tissue is vascularized, and the SWN cell lines are not myelinating cultures. Schwann cells in culture initiate a myelination program when co-cultured with neurons under precise myelination media conditions [8]. The upregulated genes were mitosis-related. This was also not unexpected as the parental tumor RNA was extracted from frozen tissue and the RNA from the cell lines are proliferating in culture.

The immortalized human SWN cell lines and normal primary human Schwann cells showed a similar rate of growth. SV40 immortalization, a widely accepted method for creating cell lines has recently raised a few concerns among the scientific community. SV40 uses blockage of the p53 and the retinoblastoma gene (RB1B) pathways to overcome cell cycle arrest. There is a concern that SV40 immortalization can create cells that are not representative of the parent tissue and are increasingly tumorigenic. Many genes have been shown to be affected by SV40 [11]. In our SV40 immortalized SWN cell lines, Cyclin A2, the Minichromosome Maintenance Complex Component genes MCM2, MCM4, MCM6, MCM7, MCM10, and Thymidine Kinase (TK1) are all upregulated when compared to parent tissue. These genes are not differentially expressed, however, when compared to non-immortalized normal human Schwann cells in culture. The growth rate of SV40 immortalized SWN cell lines is also the same as that of primary normal human Schwann cells. Therefore we suspect that the differential expression of these genes is not a peculiar consequence of our use of the SV40 virus, but is rather a function of simple growth and passaging of cells in culture. Proliferation of Schwann cells in culture requires continuous exposure to heregulin/neuregulin and forskolin [12]. Heregulin/neuregulin activates ErbB receptors and kinases, while forskolin is a cAMP elevating agent. We have maintained all of our Schwann cell cultures, both primary normal human Schwann cells and SWN cells with both neuregulin and forskolin to promote cell proliferation. It has been shown by microarray analysis of Schwann cells in culture that heregulin and forskolin upregulate 140 genes, including cyclins (cyclinB, cyclin D3, cyclin E) and other genes related to cell cycle regulation [13]. Therefore, upregulation of these genes in cells in culture may be influenced by factors needed for Schwann cell growth in culture. When performing drug screening on Schwannomatosis cells in culture, these factors influence experimental results and should be considered when choosing candidate compounds for further analysis.

In conclusion, we have created the first immortalized Schwannomatosis cell lines that retain essential genotypes, phenotypes, and cell growth patterns. These cell lines will be invaluable tools for advancing research in schwannomatosis.

## Materials and Methods

### Tissue Specimens

Schwannomatosis tumors were collected from surgical cases from December 2012 through October 2014, occurring at Johns Hopkins School of Medicine. Schwannoma diagnosis was confirmed by Pathology. This study was approved by the Institutional Review Board of the Johns Hopkins School of Medicine. After collection, the tumor specimen was placed in saline for transport to the lab. In a laminar flow hood, a section of the tumor (~0.5 cm^3^) was finely minced. The minced tumor was placed in into a new sterile 15mL conical tube containing 4ml L-15 media, and 10mg/ml collagenase/dispase. The tumor was incubated for 3 hours at 37°C to dissociate the cells. The cells were collected by centrifugation at 1,000 rpm for 2 min and the cell pellet was washed twice with 5ml of L-15 media. After the final wash the cell pellet was resuspended in in 1 mL of L15 and DNase solution (10mg/ml). The suspended cells were passed through a 70 uM cell strainer, to remove unwanted debris. The strainer was washed with an additional 3ml of L-15/DNase solution to increase cell yield. The collected cells were centrifuged at 1,000 rpm for 10 minutes. The cell pellet was suspended in 1mL of the human Schwann cell medium (Sciencell). The cells were plated in a poly-l-lysine coated T25 flask. 2–3 days after initial plating the media on the cells was changed to high glucose DMEM/pen strep, plus 10% fetal bovine serum, 2μM forskolin, and 20ng/ml neuregulin. Cultures were maintained in this media until the reached 80% confluence, at which time the cells were split into three T-25 flasks or frozen (in DMEM +20% FBS+ 10% DMSO) for later use.

### Cell Immortalization

Cells were immortalized using pLenti-SV40 from ABM (catalog number G203) according to manufacturer’s instructions. In brief, cells at passage 3 were infected with 2 ml/well of viral supernatant in the presence of 5μg/ml Polybrene, in the morning. 6–8 hours later, the viral supernatant from first infection was removed from the wells and the cells were re-infected with 2 ml/well of fresh supernatant/ polybrene. The next day, viral supernatant was removed and replaced with complete growth medium. The cells were incubated at 37°C for ~72 hours incubation and subcultured. After subculturing the cells were screened for the SV40 transgene by qRT-PCR as per manufacturer’s instructions (www.abmgood.com/SV40-Cell-Immortalization.html#8) SV40 Forward Primer Sequence 5’ ACTGAGGGGCCTGAAATGA; SV40 Reverse Primer Sequence 5’ GACTCAGGGCATGAAACAGG. The product size is 61 bp.

### Microarray

RNA from SWN tumor samples, SWN cell lines, and normal primary Human Schwann cells were analyzed using the Illumina Human HT-12 microarray covering more than 47,000 transcripts and known splice variants. Differences in expression between the two groups were identified using the Ingenuity iReport software, and Nexus Gene expression software. Genes that are upregulated or downregulated at least 2 fold and p<0.05 were considered differentially expression.

#### RNA extraction

Tumor tissues were homogenized using the polytron PT1300 tissue homogenizer followed by additional homogenization using a Qiashredder spin column (Qiagen). RNA DNA and protein were extracted using the Allprep extraction kit (Qiagen). RNA from cells in culture was extracted using Trizol, followed by clean up with RNeasy RNA extraction kit (Qiagen).

#### cDNA

1ug of total RNA was converted to cDNA using the Quantitect Reverse Transcription kit (Qiagen).

#### qPCR Primers

B2M, GAPDH, SMARCB1, p75/NGFR, NGF, S100B, CCNA2, CCNB1, CCNB2, CCND1, CCNF, and CDC20 were purchased from Qiagen. Amplified bands were analyzed for correct size and sequenced by the Johns Hopkins Sequencing core to assure correct targets.

#### qPCR

One microliter of each tumor, cell line, and normal primary human Schwann cell cDNA, was used for real-time qRT-PCR using QuantiTect SYBR Green PCR kit (Qiagen). Amplifications were carried out in 96 well plates in a Roche Lightcycler. All reactions were performed in triplicate. Dissociation curve analysis was performed to rule out experimental PCR artifacts or non-specific amplification. Expression of genes relative to Beta-2-Microglobulin (B2M) or glyceraldehyde-3-phosphate dehydrogenase (GAPDH) was calculated based on the threshold cycle (C_t_) as 2^−Δ(ΔCt)^, where ^Δ^C_t_ = C_t,_ *GENE* − C_t_ housekeeping genes and ^Δ^(^Δ^C_t_) = ^Δ^C_t,C_− ^Δ^C_t,pt_ (*C*, *cells*, *PT*, parent tumor.)

### Data analysis and Statistics

#### GO analysis

Functional gene groupings were complied using DAVID gene ontology software www.david.abcc.ncifcrf.gov

### qRT-PCR

Fold change in gene expression was determined by the 2^(- Delta Delta Ct) method following the guidelines of Zhang and Ruschhaupt 2013 www.bioconductor.org. Statistical significance of the RT-PCR data was determined using an unpaired t-test. www.graphpad.com. Standard error of mean was also calculated for the control and test groups. Standard error of the mean equals standard deviation/ square root of the sample size. SE = SD/√n

## Supporting Information

S1 Fig STR profiling of cell line Hp-SWN-14F. Authentication of the cell line using Short Tandem Repeat (STR) profiling was employed to ensure the proper identity of the subsequent passages.
(DOCX)

S1 Table The Illumina HT-12 microarray gene expression analysis comparing parent tumor and cell line gene expression. (XLSX)
