# Supplementary figures and images for: Immortalized Human Schwann Cell Lines Derived From Tumors of Schwannomatosis Patients
Source: PLoS One. 2015 Dec 14;10(12):e0144620. doi: 10.1371/journal.pone.0144620 (PMC4682832; doi:10.1371/journal.pone.0144620)

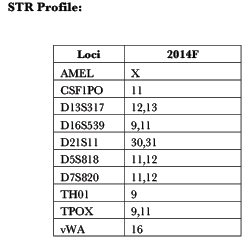


Supp Fig 1: STR profiling of SWN cell line Hp-SWN-14F

Supplement: S1 Fig — Authentication of the cell line using Short Tandem Repeat (STR) profiling was employed to ensure the proper identity of the subsequent passages. (DOCX) [file pone.0144620.s001.docx]
